# Phylogeography and domestication of Indian river buffalo

**DOI:** 10.1186/1471-2148-7-186

**Published:** 2007-10-04

**Authors:** Satish Kumar, Muniyandi Nagarajan, Jasmeet S Sandhu, Niraj Kumar, Vandana Behl

**Affiliations:** 1 Centre for Cellular and Molecular Biology, Uppal Road, Hyderabad-500007, India

## Abstract

**Background:**

The water buffalo- *Bubalus bubalis *holds tremendous potential in livestock sector in many Asian countries, particularly India. The origin, domestication and genetic structure of the Indian river buffalo are poorly understood. Therefore, to understand the relationship among the maternal lineages of Indian river buffalo breeds and their domestication process, we analysed mitochondrial D-loop region of 217 animals representing eight breeds from eight different locations in India along with published sequences of Mediterranean buffalo.

**Results:**

The maximum parsimony tree showed one major clade with six internal branches. Reduced median network revealed expansion from more than one set of haplotypes indicating complex domestication events for this species. In addition, we found several singleton haplotypes. Using rho statistics, we obtained a time estimate of 6300 years BP for the expansion of one set of hapltoypes of the Indian domestic buffalo. A few breed specific branches in the network indicated an ancient time depth of differentiation of some of the maternal lineages of river buffalo breeds. The multidimensional display of breed pairwise F_ST _values showed significant breed differentiation.

**Conclusion:**

Present day river buffalo is the result of complex domestication processes involving more than one maternal lineage and a significant maternal gene flow from the wild populations after the initial domestication events. Our data are consistent with the available archaeological information in supporting the proposition that the river buffalo was likely to be domesticated in the Western region of the Indian subcontinent, specifically the present day breeding tracts of the Mehsana, Surati and Pandharpuri breeds.

## Background

The domestic water buffalo is a major source of meat, draught, hide and employment to the marginal farmers and landless labourers in many Asian countries. Broadly, water buffalo is of two types: 1) River buffalo- distributed in the Indian sub-continent, Middle-east and Eastern Europe. 2) Swamp buffalo- distributed in North-eastern parts of India, Bangladesh, China and South-east Asian countries [1]. India has approximately ninety-eight million buffalo, primarily of the river type, representing fifty-six percent of the total world population. The importance of this species to the Indian dairy industry is immense; buffalo constitute 35% percent of the bovine population in India but they contribute more than 55% to the total milk production. There are at least nine well-defined breeds in India, representing approximately 30% of the total buffalo population of 90 million; the remaining animals are non-descript [2].

Mitochondrial genome is inherited through maternal lineage only, and unlike the nuclear genome there is a complete absence of genetic recombination in mtDNA. These two factors along with the comparative ease of genotyping of large number of individuals have meant that mtDNA polymorphisms have found tremendous usage in the evolutionary studies of maternal lineages of various species. Five to ten fold higher mutation rate of mtDNA control region as compared to that of single copy nuclear genes makes mtDNA control region extremely suitable for studying the process of derivation of domesticated animal stocks from their respective wild ancestors. Mitochondrial D-loop DNA sequences have provided significant insights into the domestication and past migration history of cattle [3-5], sheep [6], goat [7,8] and pig [9]. It has become increasingly clear that most of the livestock species have been domesticated more than once [5,7,10,11]. Recently, it has been shown that the Indian river buffalo has been domesticated independent of the swamp buffalo [12,13]. To analyse the population structure of the Indian river buffalo, and to understand the process of their domestication, we have analysed the mitochondrial D-loop region of 217 animals from the eight different breeds sampled from eight locations in India. We have also included in our analysis the published mtDNA D-loop sequences of the Mediterranean buffalo.

## Results

### Mitochondrial DNA variation in Indian river buffalo breeds

The comparison of mtDNA D-loop region (954 bp) of eight Indian river buffalo breeds revealed 135 different mitochondrial haplotypes resulting from seventy-four variable sites. The ratio of transitions: transversions was high (17:1), revealing a strong transition bias in the domestic river buffalo, as has also been observed for cattle [4] and goats [7,8]. Haplotype diversity values ranged from 0.940 ± 0.020 in the Toda to 0.997 ± 0.011 in the Pandharpuri breed (Table 1). To find out the breed effect on mtDNA sequence variation, we performed AMOVA analysis. Breed was a significant source of variation (*P *< 0.01) contributing to 10.54% of the total variation; the remaining 89.46% of variation was within breeds. The variation in the D-loop DNA sequences did not provide any support to the grouping of these breeds based upon geographical and morphological criteria [1], since only a small fraction of the total variation was attributed to between-groups variation component. However, classification of the Toda breed as one group, and the remaining seven breeds into a second group explained 8.83% (*P *< 0.01) of the total variation indicating that the between-breeds variance component was mainly a result of disproportionate contribution from the Toda.

**Table 1:** Haplotype diversity of eight Indian buffalo breeds.

Breed	Sample Size	Number of Haplotypes	Diversity
Murrah	30	19	0.9563 ± 0.0213
Bhadawari	31	19	0.9570 ± 0.0200
Mehsana	27	25	0.9943 ± 0.0119
Surati	29	25	0.9901 ± 0.0116
Jaffarabadi	21	16	0.9619 ± 0.0302
Nagpuri	22	16	0.9610 ± 0.0278
Pandharpuri	27	26	0.9972 ± 0.0111
Toda	30	15	0.9402 ± 0.0202

**Figure 1:**
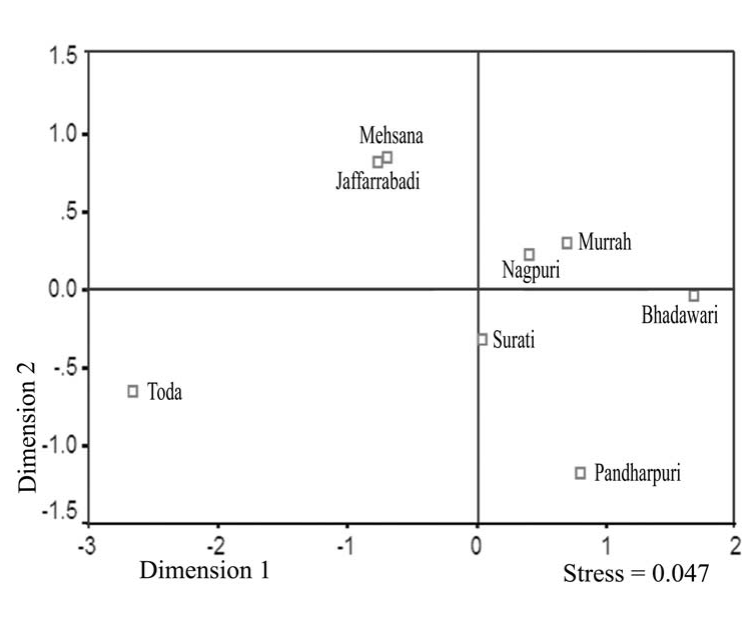
**A multidimensional scaling plot (MDS)**. The MDS plot drawn using pairwise F_ST _values of eight Indian river buffalo breeds showing genetic relationship among them.

**Figure 2:**
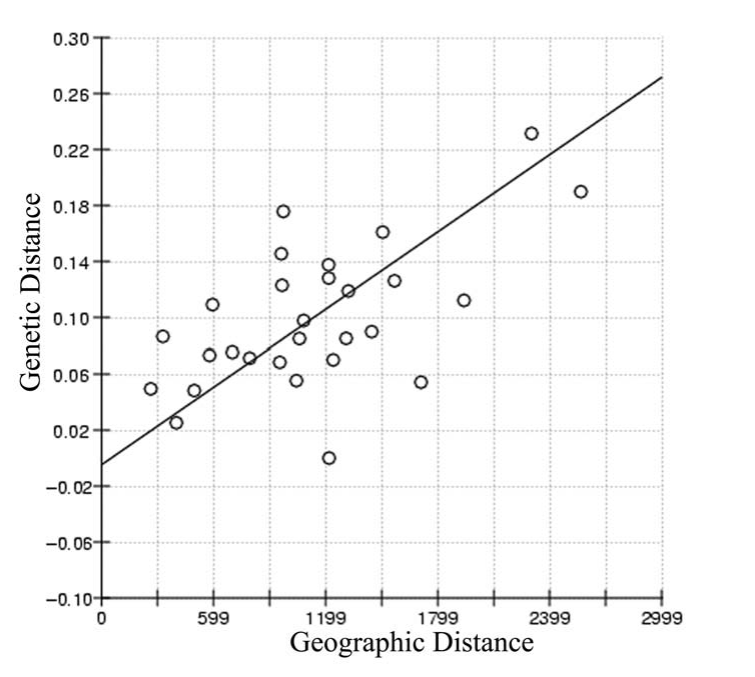
**Isolation by distance relationship**. Scatter plot of pairwise F_ST _*vs *geographic distances of eight Indian river buffalo breeds showing significant correlation between geographic and genetic distance.

To further investigate the breed differentiation, F_ST _values were calculated for all the possible breed pairs. No differentiation was observed between the Mehsana-Jaffarabadi and Murrah-Nagpuri pairs and maximum differentiation was found between the Toda and Bhadawari breeds (23.2 %). Multidimensional scaling display of pair wise F_ST _values revealed a strong evidence for structuring among these breeds with a stress value of 0.047 (Fig. 1). The eight river breeds included in our study appeared to form five clusters. We checked whether mitochondrial data set showed equilibrium with respect to change in diversity through drift and migration. A significant positive correlation (r = 0.59, *P *< 0.02) between the geographical distance and corresponding F_ST _value for the various breed pairs indicated that migration and drift were in equilibrium (Fig. 2). Our earlier study using microsatellite genotyping data indicated sub-structuring in the Pandharpuri breed [14]. Therefore, we repeated our IBD analysis with D-loop DNA sequences after removing the Pandharpuri breed from our analysis and we obtained a higher correlation co-efficient (r = 0.7259, *P *< 0.007).

### Phylogenetic analysis

**Figure 3:**
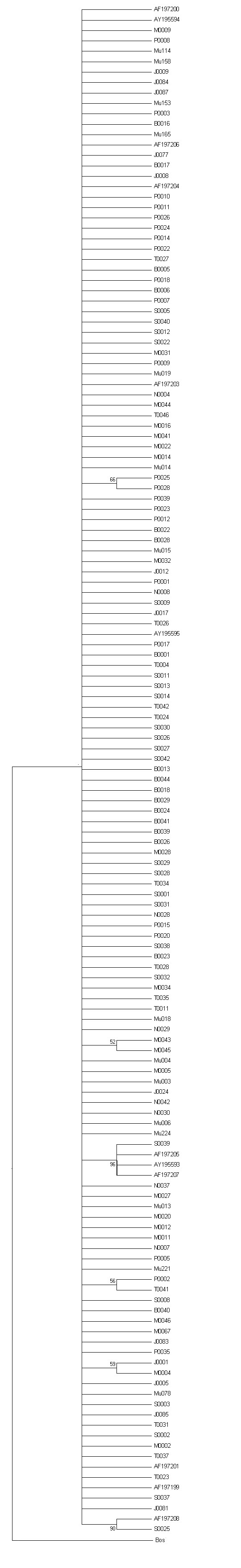
**Maximum parsimony tree of river buffalo with *Bos taurus *as an outgroup**. Numbers above the branches correspond to the bootstrap using 1000 sequence replicates.

To understand the population structure of the Indian river buffalo, we constructed the maximum parsimony tree using 135 haplotypes of the Indian river buffalo, and 11 haplotypes of the Mediterranean buffalo based upon 945 bp mtDNA sequences. Figure 3 shows the maximum parsimony tree, rooted by the *Bos taurus *sequence. This tree showed one major clade and six internal branches. The major clade encompassed the maximum number of haplotypes of the Indian and Mediterranean buffalo. The Mediterranean haplotypes intermingled with the Indian buffalo haplotypes. It may be noted that the Mediterranean haplotypes were distributed in three of the seven branches representing the Indian river buffalo.

### Reduced median network analysis of river buffalo

**Figure 4:**
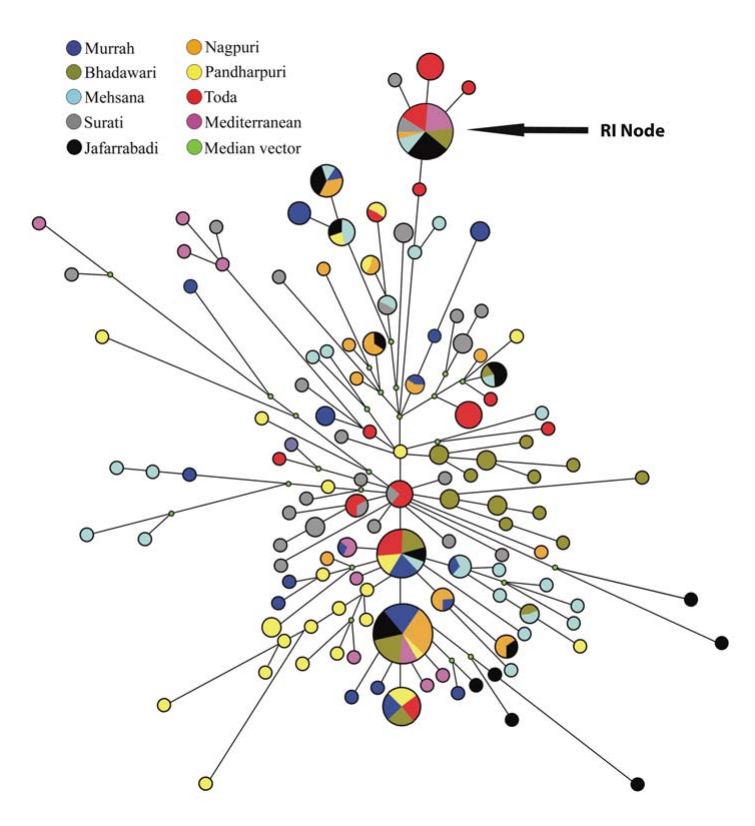
**Reduced median network of Indian and Mediterranean river buffalo**. The reduced median network was constructed using NETWORK 4.1.1.2 program with 921 bp sequences of mtDNA D-loop region of the Indian and Mediterranean river buffalo. The size of the circle is proportional to the number of animals represented. The length of the line represents the number of mutational steps.

Reduced median network based upon a 921 bp sequence (Fig. 4) revealed the lack of a single star-like appearance of the network connecting all the present day river buffalo haplotypes. This was contrary to the typical signatures of expansion of a few seeding haplotypes after domestication, and the subsequent derivation of the present day haplotypes through mutational steps. Nevertheless, within a complex network of a plethora of singletons, there were two regions, one at each end of the network, suggesting expansion from the founding haplotypes. At one end, there were four haplotypes, interconnected through one mutational step, three of which acted as radiating nodes for a large number of haplotypes. At the other end of the network, there was an isolated node (R1) connecting twenty-five animals within one mutational step. To test the significance of this grouping, we performed AMOVA analysis by classifying 217 Indian animals into two groups, viz.; 1) twenty-five animals with one mutation step away from the node R1, and b) all the remaining Indian buffalo as a separate group. This classification explained 55.75% of the total molecular variation present in the Indian animals data. To determine whether this duality was encompassing various geographical regions, we constructed reduced median networks based on the hypervariable region I (375 bp) for each breeds (data not shown). The Mehsana network was extremely complex. In all the remaining breeds, excepting the Nagpuri from Central India, there was a general tendency towards this dichotomy. It may be noted that all animals shown on internal branches of the river buffalo clade in parsimony tree were also located distantly in the network away from the expanding haplotypes. Interestingly, majority of the individuals of the Mehsana and Surati breeds sampled from Western India, were distributed all over the network as singletons. Further, we found a few breed-specific maternal branches in the network.

### Dating of population expansion of Indian river buffalo

Reduced median network (Fig. 4) showed multiple and overlapping star-like appearances suggesting expansion of the corresponding haplotypes in the background of several singletons. The time depth of the expanding haplotypes in a reduced median network can be calculated using rho statistics [15]. To estimate the time of expansion of some of the founding haplotypes of the river buffalo, we performed reduced median network for the hypervariable region I [see Additional file 1]. In this network, many of the expanding haplotypes that might have acted as nodes were interconnected through a single mutational step, thus making it difficult to estimate the time depth of their expansion. However, we could calculate the rho statistics for the minor lineage of river buffaloes represented by the R1 node (Fig. 4 & see Additional file 1) since the latter was isolated in the network. We obtained a rho value of 0.756 (95% confidence interval: 0.582 – 0.864) resulting into an expansion time of 6300 years BP using a mutation rate of 32% per nucleotide Myr^-1^[16].

## Discussion

Both the AMOVA and MDS display of F_ST _values suggested a strong breed effect on the D-loop sequence variation. The MDS display based on the mitochondrial sequences was broadly reminiscent of the cluster structure of these breeds obtained from 27 microsatellite loci [14] with two notable exceptions. The microsatellite markers showed clustering of the Mehsana and Bhadawari breeds along with an admixture of the Murrah, Surati and Nagpuri breeds. However, in the present study the maternal lineage of the Mehsana breed stood out from the admixture and instead located itself along with the Jaffarabadi (Fig. 1). Similarly the maternal lineage of the Bhadawari breed tended to make its own cluster. There is an anecdotal evidence to suggest that the Mehsana breed has been an outcome of gene flow from the Murrah males in the recent past. That would explain one of the major differences in the clustering results obtained from the maternal and autosomal markers. Generally it is believed that the breed formation may be a very late activity with a low time depth vis-à-vis the process of domestication of a particular livestock species [11] and the breed differentiation may be the result of an intensive selection mainly operating through the paternal lineages. However, it was surprising to note strong breed effect on the mitochondrial variation (Fig. 1). A few breed specific branches in the network (Fig. 4) indicated an ancient time depth of differentiation of some of the maternal lineages of river buffalo breeds. It appears that the very concept of 'breed' in the river buffalo may not have the same connotation as in the case of modern breeds of cattle in the Western world [3,4]. These results should have obvious implications in arriving at rational decisions aimed at conservation of buffalo genetic variability [2].

The phylogenetic analysis in the present study unveiled one main clade with six internal branches for the river buffalo. The Mediterranean haplotypes intermingled with the Indian buffalo haplotypes. This is consistent with a common origin of the Mediterranean and Indian domestic river buffalo. In reduced median network there was evidence in support of expansion from four nodes, out of which one node, namely R1, was far off from the remaining three expanding nodes. There were several minor branches and a large number of singleton haplotypes located far away from these nodes. Our phylogenetic and network results clearly demonstrated that the present day river buffalo are an outcome of a complex domestication process. Multiple domestication events appear to be a rule rather than an exception in livestock species [5,10,11]. For an example, horses have been domesticated from several wild populations at different locations and a few mitochondrial lineages of modern horse population are breed- specific [10]. With the Indian buffalo, particularly the Mehsana breed, the situation is much more complicated because of the presence of several singleton haplotypes. It might be possible that a very large number of females with highly divergent mitochondrial sequences contributed to the domesticated stocks. Alternatively, there might have been a 'trickling in' effect in terms of continuous addition of females from the wild to the domestic herds over an extended period. The co-existence of the domestic and wild stocks in the same time and space, an essential requirement in favour of such a scenario, has been well known [14,17].

Our estimate of the expansion time of one of the haplotypes was 6300 years BP using rho statistics. Water buffalo has been depicted on the Indus Valley civilization seals dated around the third millennium BC. This information has been used to suggest that buffalo was probably domesticated around that time in these areas [18]. Such a proposition does not stand scrutiny. On the contrary, the form and context are more akin to these individuals being of the wild types. Wild buffalo remains have been recovered from ancient sites in Balochistan- Mehargarh in Pakistan and Santhli in North Gujarat [17]. Buffalo remains dating around mid third millennium BC from Kutch-Dholavira, Gujarat in India are thought to be from the domestic buffalo as determined from the size differences between the Dholavira and Santhli/Mehargarh remains [17]. These workers have argued that the suggested importance of milk and milk products during the Harappan times [19,20] and the presence of domestic buffalo during this period are consistent with each other, implying that water buffalo might have already been the dairy animal in the Indus Valley civilization by then. Further, on the basis of size diminution of the buffalo from Dholavira, it has been suggested that the process of domestication of water buffalo was likely to be extended earlier than the Harappan period [17]. The time estimate of expansion of at least one of the haplotypes of river buffalo (6300 years BP) in the present study is consistent with this suggestion of these authors.

**Figure 5:**
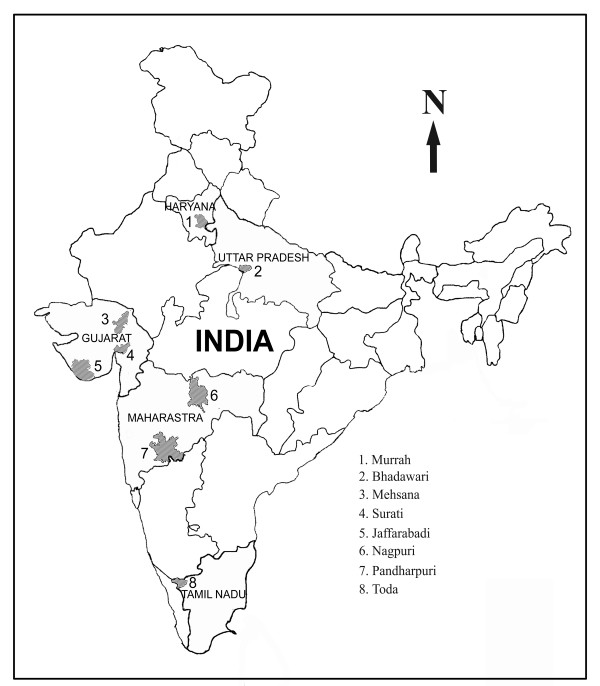
**Outline map of India**. It shows the sampling sites of the eight Indian river buffalo breeds used in this study.

The present study showed extremely high haplotype diversity in the Mehsana, Surati and Pandharpuri breeds from Western India (Fig. 5 & Table 1). It is expected that the genetic variability would be higher at the point of origin of the domesticated species. Therefore, it is reasonable to propose that if the buffalo was domesticated in the Indian subcontinent, the Western region represented by the present day breeding tract of the Mehsana, Surati and Pandharpuri breeds appears to be the most likely candidate. A closer examination of our reduced median network [see Additional file 1] indeed supported such a proposition. The network analysis revealed four nodes with an indication of expansion of the founding haplotypes in an otherwise very complex network resulting from several singletons. These nodes and additional haplotypes within two mutational steps away from any of these nodes included 140 individuals. Approximately 48% of the Mehsana, Surati and Pandharpuri animals belonged to this group. On the other hand, the individual contribution of the remaining five breeds to this group ranged from 63% in the case of the Murrah breed to 90% in the Toda breed, suggesting a decline in the D-loop haplotype complexity in these five breeds as one moved away from the sampling sites of the Mehsana/Surati/Pandharpuri animals. In this context it is interesting to note that the Western region of India is home to several recognized breeds of river buffalo encompassing extensive phenotypic [1] and microsatellite DNA variations [14]. Earlier we reported four haplotypes of *cytochrome b *gene in a sample of eighty buffalo representing different breeds [12]. Interestingly, all these four haplotypes were present between Surati and Pandharpuri animals against the presence of any two of these haplotypes in the remaining breeds. We have not examined the mitochondrial sequences of buffalo from Pakistan. It may be possible that the site(s) for domestication of buffalo may extend to the Pakistani areas.

## Conclusion

We have earlier shown that the Indian river buffalo were domesticated independent of swamp buffalo [12]. Our present study supports the following scenario of the river buffalo domestication. The Mediterranean and Indian domestic river buffalo have been derived from the same stocks through a complex process. During domestication and/or afterwards there might have been a continuous influx of maternal variability from the wild buffalo into the domestic stocks. The time of expansion of at least one of the several expanding haplotypes was estimated to be 6300 years BP. If the river buffalo was domesticated in the Indian subcontinent, as has been suggested by archaeological studies, the Western region represented by the present day breeding tract of the Mehsana, Surati and Pandharpuri breeds appears to be the most likely candidate region.

## Methods

### Sample collection, amplification and sequencing of mitochondrial DNA

Two hundred and seventeen river buffalo samples representing eight different breeds from Northern, Northwestern, Central and Southern India (Fig. 5) were used in this study. The sampling and sequencing protocol have been described in our earlier study [12].

### Sequence alignment and population analyses

AUTOASSEMBLER (Perkin Elmer) and CLUSTALX programs [21] were used for sequence editing and alignment purpose respectively. Haplotype diversity, AMOVA and population pairwise differences (F_ST_) were calculated using ARLEQUIN version 2.001 [22]. The pair wise F_ST _values were displayed by multidimensional scaling (MDS) using SPSS11.0_. _Isolation by distance (IBD) between breeds was examined by Mantel's tests [23] as implemented in IBD 2.1 program [24].

### Phylogenetic analyses

To construct phylogenetic tree, 135 D-loop haplotypes obtained from 217 Indian river buffalo sequences and 11 published haplotypes of Mediterranean buffalo were used. We considered a 945 bp fragment of the D-loop region for which sequences were available for the two types of river buffalo. *Bos taurus* (NCBI: accession no.: NC_006853) was used as the out-group. A maximum parsimony (MP) tree was constructed using MEGA 3.1 [25]. The close-neighbour-interchange algorithm was chosen with a search level of three. The searches included 100 replications of random addition trees with 1000 bootstrapping. In addition, the reduced median network was applied to the dataset containing 228 sequences of river buffalo (Indian and Mediterranean) using NETWORK 4.1.1.2 program [26] with the parameters set to a weight of two and threshold value of one.

## Competing interests

The author(s) declares that there are no competing interests.

## Authors' contributions

SK conceived and designed research; JSS, NK and VB performed experiments; MN analysed the data, and SK and MN wrote the paper.

## Supplementary Material

Additional file 1 **Reduced median network of river buffalo based on mtDNA D-loop HVRI (375 bp) sequences**. Circle size is proportional to the number of animals represented. The length of the line represents the number of mutations. Click here for file

## References

[B1] Cockrill WR (1981). The water buffalo: a review. Brit Vet J.

[B2] George M, Balaine DS, Vij PK, Kumar S, Nagarcenkar R (1988). Conservation and management of buffalo genetic resources of India. Buffalo Production and Health.

[B3] Loftus RT, MacHugh DE, Bradley DG, Sharp PM, Cunningham P (1994). Evidence for two independent domestications of cattle. Proc Natl Acad Sci USA.

[B4] Bradley DG, MacHugh DE, Cunningham P, Loftus RT (1996). Mitochondrial diversity and the origin of the African and European cattle. Proc Natl Acad Sci USA.

[B5] Beja-Pereira A, Caramelli D, Lalueza-Fox C, Vernesi C, Ferrand N, Casoli A, Goyache F, Royo LJ, Conti S, Lari M, Martini A, Ouragh L, Magid A, Atash A, Zsolnai A, Boscato P, Triantaphylidis C, Ploumi K, Sineo L, Mallegni F, Taberlet P, Erhardt G, Sampietro L, Bertranpetit J, Barbujani G, Luikart G, Bertorelle G (2006). The origin of European cattle: evidence from modern and ancient DNA. Proc Natl Acad Sci USA.

[B6] Loehr J, Worley K, Grapputo A, Carey J, Veitch A, Coltman DW (2006). Evidence for cryptic glacial refugia from North American mountain sheep mitochondrial DNA. J Evol Biol.

[B7] Luikart G, Giellly L, Excoffier L, Vigne JD, Bouuvet J, Taberlet P (2001). Multiple maternal origins and weak phylogeographic structure in domestic goats. Proc Natl Acad Sci USA.

[B8] Joshi MB, Rout PK, Mandal AK, Smith CT, Singh L, Thangaraj K (2004). Phylogeography and origin of Indian domestic goats. Mol Biol Evol.

[B9] Giuffra E, Kijas JMH, Amarger V, Carlborg O, Jeon JT, Andersson L (2000). The Origin of the domestic pig: Independent domestication and subsequent introgression. Genetics.

[B10] Jansen T, Forster P, Levine MA, Oelke H, Hurles M, Renfrew C, Weber J, Olek K (2002). Mitochondrial DNA and the origins of the domestic horse. Proc Natl Acad Sci USA.

[B11] Bruford MW, Bradley DG, Luikart G (2003). DNA markers reveal the complexity of livestock domestication. Nat Rev Genet.

[B12] Kumar S, Nagarajan M, Sandhu JS, Kumar N, Behl V, Nishanth G (2007). Mitochondrial DNA analyses of Indian water buffalo support a distinct genetic origin of river and swamp buffalo. Anim Genet.

[B13] Lei CZ, Zhang W, Chen H, Lu F, Liu RY, Yang XY, Zhang HC, Liu ZG, Yao LB, Lu ZF, Zhao ZL (2007). Independent maternal origin of Chinese swamp buffalo (*Bubalus bubalis*). Anim Genet.

[B14] Kumar S, Gupta J, Kumar N, Dikshit K, Navani N, Jain P, Nagarajan M (2006). Genetic variation and relationships among eight Indian riverine buffalo breeds. Mol Ecol.

[B15] Forster P, Harding R, Torroni A, Bandelt AJ (1996). Origin and evolution of native American mtDNA variation: A reappraisal. Am J Hum Genet.

[B16] Shapiro B, Drummond AJ, Rambaut A, Wilson MC, Matheus PE, Sher AV, Pybus OG, Gilbert MTP, Barnes I, Binladen J, Willerslev E, Hansen AJ, Baryshnikov GF, Burns JA, Davydov S, Driver JC, Froese DG, Harington CR, Keddie G, Kosintsev P, Kunz ML, Martin LD, Stephenson RO, Storer J, Tedford R, Zimov S, Cooper A (2004). Rise and fall of the beringian steppe bison. Science.

[B17] Patel AK, Meadow RH, Buitenhuis HL, Bartosiewicz L, Choyke AM (1998). The exploitation of wild and domestic water buffalo in prehistoric northwestern south Asia. Archaeology of the near east: Proceedings of the third international symposium on the archeozoology of the southwestern Asia and adjacent areas.

[B18] Zeuner FE (1963). A history of domesticated animals. London, Hutchinson.

[B19] Gouin P (1990). Rapes, Jarres et faisselles: la production et l'exploration des produits laitiers dans l'Indus du 3e millenaire. Paleorient.

[B20] Gouin P (1992). Cuillers harappeennes: technologie et interpretation. Paleorient.

[B21] Thompson JD, Gibson TJ, Plewniak F, Jeanmougin F, Higgins DG (1997). The ClustalX windows interface: flexible strategies for multiple sequence alignment aided by quality analysis tools. Nucleic Acids Res.

[B22] Schneider S, Roessli D, Excoffier L (2002). ARLEQUIN 2.0: A software for population genetic data analysis. Genetics and Biometry Laboratory, University of Geneva, Switzerland.

[B23] Mantel NA (1967). The detection of disease clustering and a generalized regression approach. Cancer Res.

[B24] Jensen JA, Bohonak J, Kelley SK (2005). Isolation by distance, web service. BMC Genet.

[B25] Kumar S, Tamura K, Nei M (2004). MEGA 3.1: Integrated software for molecular evolutionary genetics analysis and sequence alignment. Brief Bioinform.

[B26] Bandelt HJ, Forster P, Sykes BC, Richards MB (1995). Mitochondrial portraits of human populations. Genetics.

