# Supplementary figures and images for: Phylogeography and domestication of Indian river buffalo
Source: BMC Evol Biol. 2007 Oct 4;7:186. doi: 10.1186/1471-2148-7-186 (PMC2140268; doi:10.1186/1471-2148-7-186)

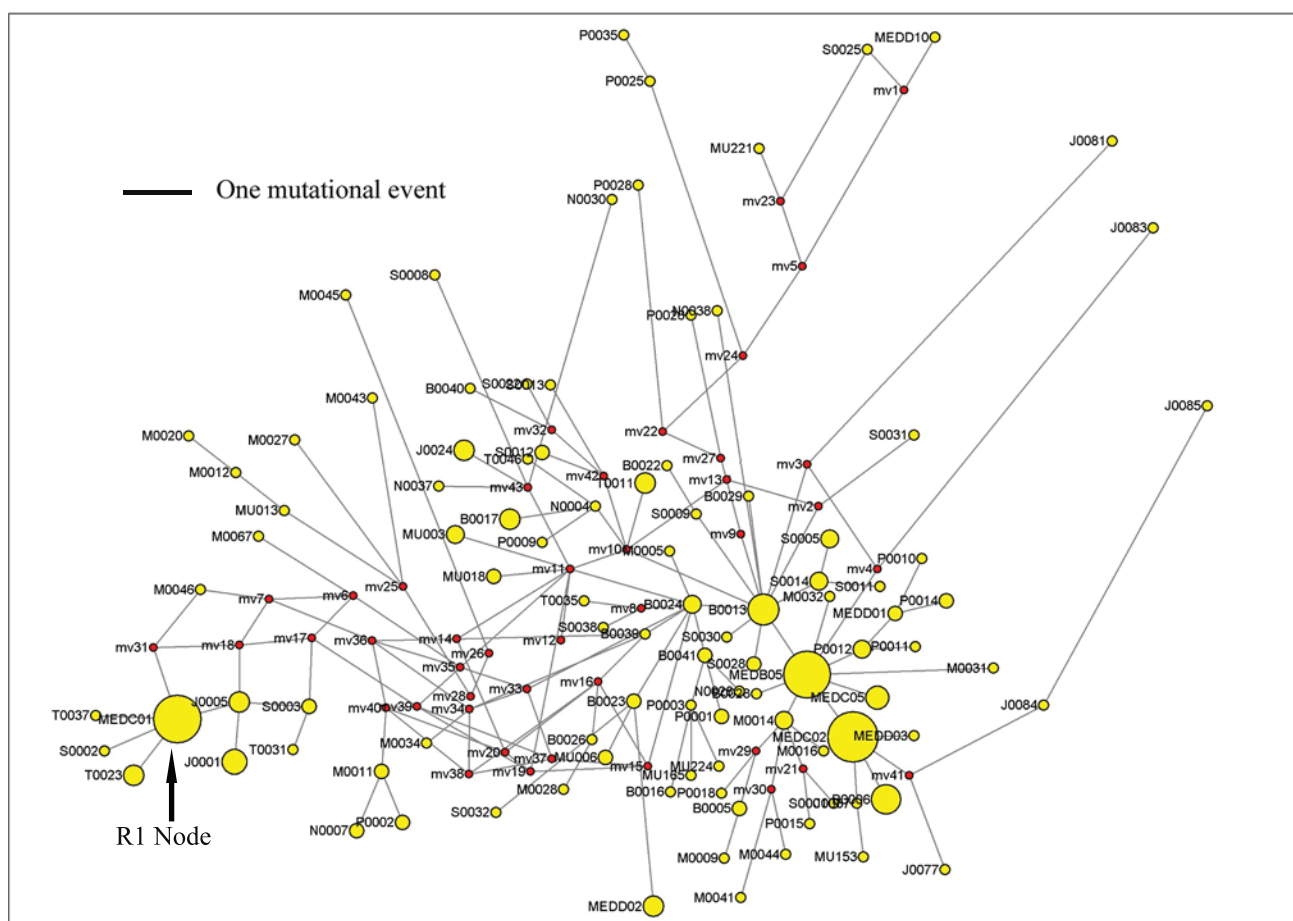

Supplement: Additional file 1 — Reduced median network of river buffalo based on mtDNA D-loop HVRI (375 bp) sequences. Circle size is proportional to the number of animals represented. The length of the line represents the number of mutations. [file 1471-2148-7-186-S1.pdf]
